# Ambulances for London

**Published:** 1904-03-05

**Authors:** 


### AMBULANCES FOR LONDON.

THE following letter has been issued by the Hospitals Association to the daily Press:—

The question of an efficient ambulance service for London, with which the Hospitals Association has been actively identified for many years, has, owing to the apparent futility of the recent London County Council inquiry, attracted a good deal of public attention lately, and is of such great public importance that energetic steps should be taken to deal with it.

Having for upwards of 12 years maintained some 60 ambulances in London, the council of this Association have an extensive practical acquaintance with Metropolitan requirements; and, believing that the prospect of those requirements being fully and expeditiously met depends largely on whether the matter is taken up by the London County Council, the Association has decided to briefly make known their views to those gentlemen whose candidature for seats on the Council is now before the electors.

We will confine our remarks to that branch of the subject which relates to provision for cases of accident or sudden illness in the streets, and which is much the most important.

We record our belief that an elaborate scheme and an extensive organisation involving a large expenditure are not called for. We are convinced that what is wrong can be defined with precision, and that the remedies are obvious, simple and inexpensive.

In the first place it is essential to recognise that effective provision for a case of street accident consists:—

1. Of efficient first aid to the sufferer on the spot.
2. Of proper means of transport for cases whose removal to hospital or elsewhere is necessary.

Of the two, the first is the more important, and yet in the proposals that have recently been made from various quarters it has been entirely overlooked, and the sole cry has been "horsed ambulances." Quickness of transport has monopolised attention, and the question of prompt and skilful "first-aid," which is of much greater moment, has been lost sight of.

It is difficult to exaggerate the mischief which may be caused by the unskilful handling of injured persons, in the first stage, before the question arises of transport at all. It is then that the stopping of bleeding, or the splinting of a broken limb, may mean the saving of life or the prevention of much suffering.

The next point is that this most important duty falls, in the great majority of cases, to the lot of the police. This is a fact of very great importance, because the inevitable conclusion from it is that it is absolutely necessary that the police be well trained and be kept efficient in first aid.

What do we find? It was stated in evidence before the London County Council sub-committee that "the police are not sufficiently trained, one short course of but five lectures being in the case of some of the men considered sufficient." Sir W. J. Collins in his prefatory note to the report of the sub-committee, writes: "Opportunity is afforded to constables for instruction in first-aid." Not that instruction and maintenance in efficiency are compulsory in every case, but that "opportunity is afforded," and we have heard that the men, if they wish to take advantage of the opportunity, must do so in their own time.

This is not as it should be. It is as necessary for a policeman to be efficient in "first-aid" as it is for a soldier to be efficient in the use of his arms. What would be said of an army which merely "afforded opportunity for instruction" in rifle drill and sword exercise ?

If the first handling of cases of street accident has to be done for the most part by the police, the adequate training for it must be a peremptory necessity. If that training obtained at present, the cab as a means of transport would be a good deal less in evidence than it now is, and there would be fewer complaints about London's lack of ambulance provision.

We are aware that the control of the police is not in the hands of the County Council, but we feel that if the above conclusions, from which it appears to us there is no escape, meet with the recognition of the members of the Council, their influence will soon bring about the desired reform.

We now pass on to the question of the means of transport. In the first place it is desirable to know how London now stands in this matter. If, with Charing Cross as centre, a circle be described with a radius of 2 miles, that is comprising an area of upwards of 12 square miles extending from Camden Town on the north to Kennington Oval on the south, and from South Kensington on the west to Aldgate on the east, we find that there are 26 Bischoffsheim ambulances stationed within it, and that there is scarcely a spot inside the circle which is more than half a mile from one of them. Stated in terms of minutes, instead of miles, there is no spot where the ambulance cannot, in present conditions, be at the sufferer's side in 15 minutes, and with regard to the greater part of the area in question, 7½ minutes would be the maximum. Moreover, as the litters are placed in many cases in the busiest thoroughfares, where most accidents happen, in a large number of cases the ambulance is close at hand.

It should also be mentioned that there are 33 police-stations within the area, most of them with a wheeled ambulance, and some of these are quite suitable for invalid transport. The circle also includes 12 general hospitals. It should further be observed that it is within this area that accidents are most numerous.

We think that on the whole in this district there is not much that requires to be done; certainly there is no urgent necessity. If the ambulance were at once sent for, it would in the majority of cases be at the sufferer's side before first-aid treatment was completed, and in no case would there be much delay. In this area the fault is not so much the scarcity of ambulances but the neglect to use them in preference to cabs, which reflects on the "first-aid" training of the police as already indicated.

Outside this area the Association's ambulances are not so numerous. Moreover, the distance to hospital is much greater. It is, therefore, in the outer ring that provision for transport most needs to be supplemented, and that horse or motor ambulances are likely to be most serviceable.

The places where accidents are most frequent are—North, Highgate; East, Whitechapel and Bow; South, Wandsworth, Lambeth, Southwark, and Camberwell; West, Hammersmith.

Of these, Whitechapel and Bow are perhaps least urgent, as the Association's ambulances are more numerous in that direction. The most pressing need is undoubtedly on the south side of the river, and there is the place to try horse or motor ambulances. One in the neighbourhood of Guy's Hospital and one near St. Thomas's Hospital could not fail to be of great service, and the experience gained there would be a guide in considering the provision necessary for other parts of London.

In order of urgency the things requiring to be done are therefore:—

1. Improvement in the "first-aid" training of the police.

2. Increased provision of ambulances in South London, including a trial of horse or motor ambulances.

3. Increased provision of ambulances in North, East, and West London.

4. Rearrangement of some stations in the central area. Better sites should be found for several, and a few stations might be added.

Item 1 is a matter that this Association can do nothing practical to forward. The police have now to do the work, and as far as we can see, must for obvious reasons have to do it in the future. Public opinion when it recognises the fact, will soon bring about the compulsory training of the police. The members of the London County Council can do much to influence public opinion.

With regard to Item 2, we have Mr. H. L. Bischoffsheim's authority to say that if the London County Council will provide stations in the positions indicated in South London he will provide horse or motor ambulances there in order to test the value of such appliances in parts where long distances have to be covered in conveying a patient to hospital.

Items 3 and 4. Mr. Bischoffsheim is ready, if sites in suitable situations are provided, to supplement the number of wheeled litters now in the outer ring and to improve the utility of those in the two-mile circle by moving several into more busy and readily accessible situations.

